# Construction of an enantiopure bivalent nicotine vaccine using synthetic peptides

**DOI:** 10.1371/journal.pone.0178835

**Published:** 2017-06-01

**Authors:** David F. Zeigler, Richard Roque, Christopher H. Clegg

**Affiliations:** TRIA Bioscience Corp, Seattle, WA, United States of America; Instituto Butantan, BRAZIL

## Abstract

Clinical outcomes of anti-nicotine vaccines may be improved through enhancements in serum antibody affinity and concentration. Two strategies were explored to improve vaccine efficacy in outbred mice: the use of enantiopure haptens and formulation of a bivalent vaccine. Vaccines incorporating natural (-) nicotine haptens improved relative antibody affinities >10-fold over (+) haptens, stimulated a two-fold boost in nicotine serum binding capacity, and following injection with 3 cigarette equivalents of nicotine, prevented a larger proportion of nicotine (>85%) from reaching the brain. The activity of a bivalent vaccine containing (-) 3’AmNic and (-) 1’SNic haptens was then compared to dose-matched monovalent groups. It was confirmed that antisera generated by these structurally distinct haptens have minimal cross-reactivity and stimulate different B cell populations. Equivalent antibody affinities were detected between the three groups, but the bivalent group showed two-fold higher titers and an additive increase in nicotine serum binding capacity as compared to the monovalent groups. Mice immunized with the bivalent formulation also performed better in a nicotine challenge experiment, and prevented >85% of a nicotine dose equivalent to 12 cigarettes from reaching the brain. Overall, enantiopure conjugate vaccines appear to improve serum antibody affinity, while multivalent formulations increase total antibody concentration. These findings may help improve the performance of future clinical candidate vaccines.

## Introduction

Tobacco creates an undue burden on the health care systems of the world. Smoking is the leading cause of preventable mortality and morbidity in the United States. Overall, 1 in 5 deaths are caused by smoking [1]. While 50% of smokers attempt to quit annually, less than 5% are able to successfully quit on the long-term each year [2,3]. Currently, cessation aids consist of nicotine replacement therapy, pharmaceutical (ant)agonists or behavioral therapy; compared to abstinence, these increase cessation rates only modestly and can have significant side effects [4–6].

Anti-addiction vaccines are one possible solution to these problems [7–17]. Typically, these contain drug analogs that are covalently bound to a recombinant protein that provides T cell-mediated B cell help. The resultant antibodies (Abs) bind the drug as it enters the bloodstream and limit the subsequent pharmacological effects of the drug. Anti-nicotine vaccines have been tested in humans but ensuing Ab responses were inferior and much more variable than what has been observed in animals [18]. Despite this, subgroup analyses in two Phase II studies indicated that subjects with the highest Ab titers achieved 12 months of abstinence, that the non-abstaining subjects within the high Ab group reduced daily cigarette consumption by 50%, without a compensatory increase in smoking [8,19]. However, follow-on Phase III studies failed to demonstrate efficacy, with one likely explanation being that the induced anti-nicotine Ab concentration was insufficient to prevent significant nicotine entry into the brain. The failure of these first-generation vaccine studies has encouraged further research into the requirements for improving Ab affinities and concentrations, including innovations in hapten/carrier designs and adjuvants beyond Alum [8,20–36].

Previously, we described a hapten carrier made from a short alpha-helical peptide that self-assembles into a coiled-coil structure [37]. Following chemical conjugation, the carrier’s B-epitope domain contains a high density of haptens that improves antigen presentation [38–44], and its C-terminal half contains 2 universal CD4 T cell epitopes, which simultaneously activate multiple MHC Class II molecules. One advantage of this carrier is its ease of manufacturing and formulation following solid-phase protein synthesis. Additionally, it lacks non-essential but immuno-stimulatory protein sequences found in traditional carriers that induce anti-carrier Ab responses. Herein, we explore two strategies for improving Ab responses with this carrier. The first employs enantiopure (-) nicotine haptens to improve functional Ab activity [23,24]. The second utilizes a bivalent formulation with two structurally distinct haptens to increase Ab responses even further [25–28].

## Materials and methods

### Ethics statement

This study was carried out in strict accordance with the recommendations in the Guide for the Care and Use of Laboratory Animals of the National Institutes of Health, the US Public Health Service Policy on Humane Care and Use of Laboratory Animals, and the Association for Assessment and Accreditation of Laboratory Animal Care International (AAALAC). Protocol #2015–11 was approved by the Institutional Animal Care and Use Committees of the Infectious Disease Research Institute which operates under a currently approved Assurance #A4337-01 and USDA certificate #91-R-0061. Mice were housed under specific pathogen-free conditions in ventilated microisolator cages, and kept on a 12-hour light/dark schedule with free access to food and water. Animal welfare and health was monitored daily and in the rare instances where medical intervention was not effective, animals were humanely euthanized and every effort was made to minimize suffering.

### Peptides, haptens

**Fig 1:**
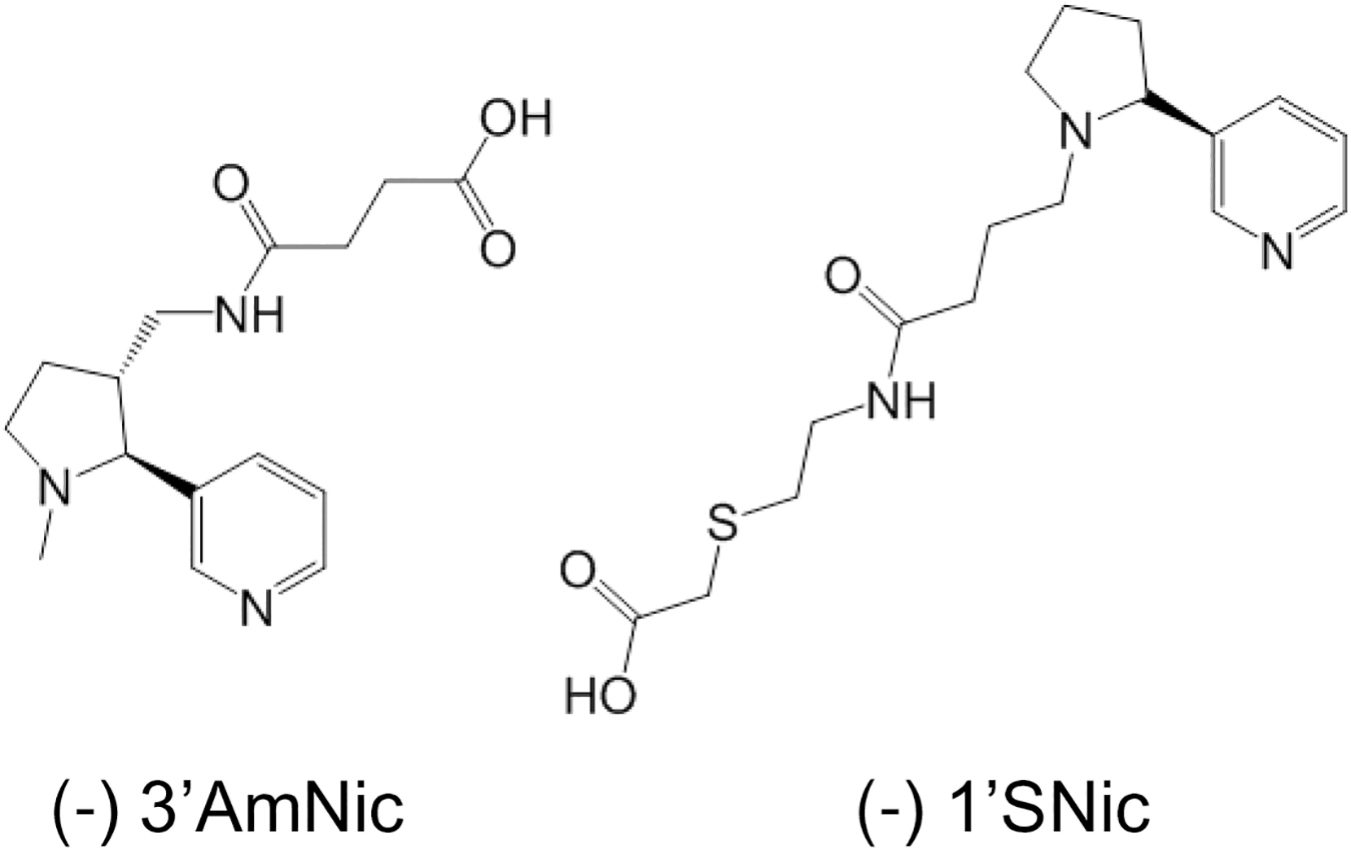
Nicotine haptens used in this study.

All peptides were synthesized at Bio-Synthesis Inc (Lewiston, TX). In addition to a coiled coil domain [37], P8 contained T cell epitopes derived from tetanus toxoid and herpes B surface antigen [45]. P9 contained epitopes from diphtheria toxoid [46] and PADRE [47]. Nicotine haptens (3’AmNic and 1’SNic, Fig 1) were synthesized (Life Chemicals, Vancouver BC) as racemic mixtures using reported methodologies [23,28]. 3’AmNic was then succinylated and 1’SNic treated with methyl bromoacetate and subsequently deprotected to the free carboxylic acid with lithium hydroxide. Enantiomer separation via supercritical fluid chromatography was performed by Averica Discovery (Marlborough, MA) and enantiomer chirality was assigned using vibrational circular dichroism (Biotools Inc., Jupiter, FL).

### Conjugations

Synthetic carrier peptide or bovine serum albumin (BSA) was dissolved in 100 mM 3-(*N*-morpholino)propanesulfonic acid **(**MOPS) buffer (50 mM NaCl, pH 7.2) at 10 mg/mL. The appropriate hapten, *N-*hydroxysuccinimide (NHS) and 3-(ethyliminomethyleneamino)-*N*,*N*-dimethylpropan-1-amine (EDC) were dissolved to 1 M in the same buffer. Hapten (400 equivalents to peptide monomer), NHS and EDC (380 equivalents to peptide monomer) were combined and agitated on a plate shaker (700 rpm, r.t.) for 30 min, after which the peptide/BSA was added to the reaction. Four hours later, the reaction was diluted to ca. 1 mg/mL with MOPS buffer and dialyzed (1K MWCO) against MOPS buffer overnight. Samples were centrifuged at 10,600 x *g* for 5 min to remove any precipitate and the hapten load was quantified as previously described [37,48]. It was determined that 100 hapten equivalents led to a loading of ~2.5 haptens/peptide monomer, while 500 equivalents yielded ~6 haptens/monomer. Loadings plateaued beyond 500 equivalents. These studies used a loading ca. 4–5 haptens/peptide monomer. To generate antigens for enzyme-linked immunosorbent assays (ELISA), BSA was conjugated with 1000 equivalents of hapten and 950 equivalents of EDC and NHS. Peptide concentrations were determined by amino acid analysis.

### Immunizations

Female CD-1 mice (Charles River Laboratories) were housed under pathogen-free conditions in the Infectious Disease Research Institute vivarium. The nicotine-conjugated peptides were combined on the day of immunization with 2% glucopyranosyl lipid adjuvant in a squalene emulsion (GLA-SE) [49]. Conjugated peptide stock solutions were diluted in GLA-SE/PBS to yield 50 µg/mL (enantiomer experiment) or 100 µg/mL (bivalent experiment) peptide conjugate. Mice were injected with 50 µL of the appropriate vaccine in each hind quadriceps muscle (100 µL total injection volume) on days 0, 21, 42 and serum was collected on days 35 and 56 for measuring nicotine-specific Ab responses.

### Antibody titers and affinities

Serum Ab titers, cross-reactivity and relative affinities were determined by ELISA as previously reported [37] with the following modifications. Serum samples were serially diluted 3-fold from 1/100 in blocking buffer. Midpoint titers at half maximal absorbance were calculated using GraphPad Prism (GraphPad Software, San Diego, CA). Ab affinities were determined as previously reported [37] with the following modifications. Nicotine was prepared at 200 mM and serially diluted to 0.2 nM in blocking buffer. 80 µL of sera (diluted to a concentration twofold higher than that which yielded half maximal titer absorbance) and nicotine were mixed in a non-absorbent 96-well plate and incubated at 37 °C for 1.5 h. Data were transformed according to the method of Friguet et al. [50] with the correction factor applied for bivalent IgG antigen binding described by Stevens et al. [51].

### Serum nicotine binding capacity

Serum was pooled from each immunization group and aliquots (100 µL) were spiked with serially diluted nicotine to achieve final nicotine concentrations of 0.01–10000 µM. These samples were then subjected to equilibrium dialysis against an equal volume of 1X phosphate-buffer saline (PBS) for 4 h (37 °C) using an HTD96b equilibrium dialysis setup (HTDialysis, Gales Ferry, CT). Aliquots from the sera and buffer sides of the dialysis membranes were removed and analyzed by liquid chromatography-tandem mass spectrometry (LC-MS/MS) (Alturas, Moscow, ID). Unbound nicotine was quantified by comparing peak intensities to an internal standard of d4-nicotine and a standard curve generated with a nicotine standard.

### Nicotine distribution in brain and sera

Anesthetized mice received a tail vein infusion (5 s) of 0.05 or 0.2 mg/kg of nicotine hydrogen tartrate diluted in 100 mL of PBS, which approximates the mg/kg dose of nicotine equivalent to three and twelve smoked cigarettes in humans, respectively [21]. Mice were sacrificed after 5 minutes. Blood was collected via cardiac puncture for serum preparation and the brain was removed, weighed and flash frozen in liquid nitrogen. Following tissue sample extraction, nicotine was measured by LC-MS/MS (Alturas Inc, Moscow ID). The amounts of nicotine detected in brain were not corrected for nicotine present in cerebral blood. Data were analyzed using GraphPad Prism. Statistical significance of the difference between two groups was calculated by Student’s 2-tailed t-test on log-transformed data and between three or more groups by 1-factor analysis of variance (ANOVA) followed by post-hoc analysis.

## Results

### Enantiopure haptens improve anti-Nicotine Ab responses in immunized mice

**Fig 2:**
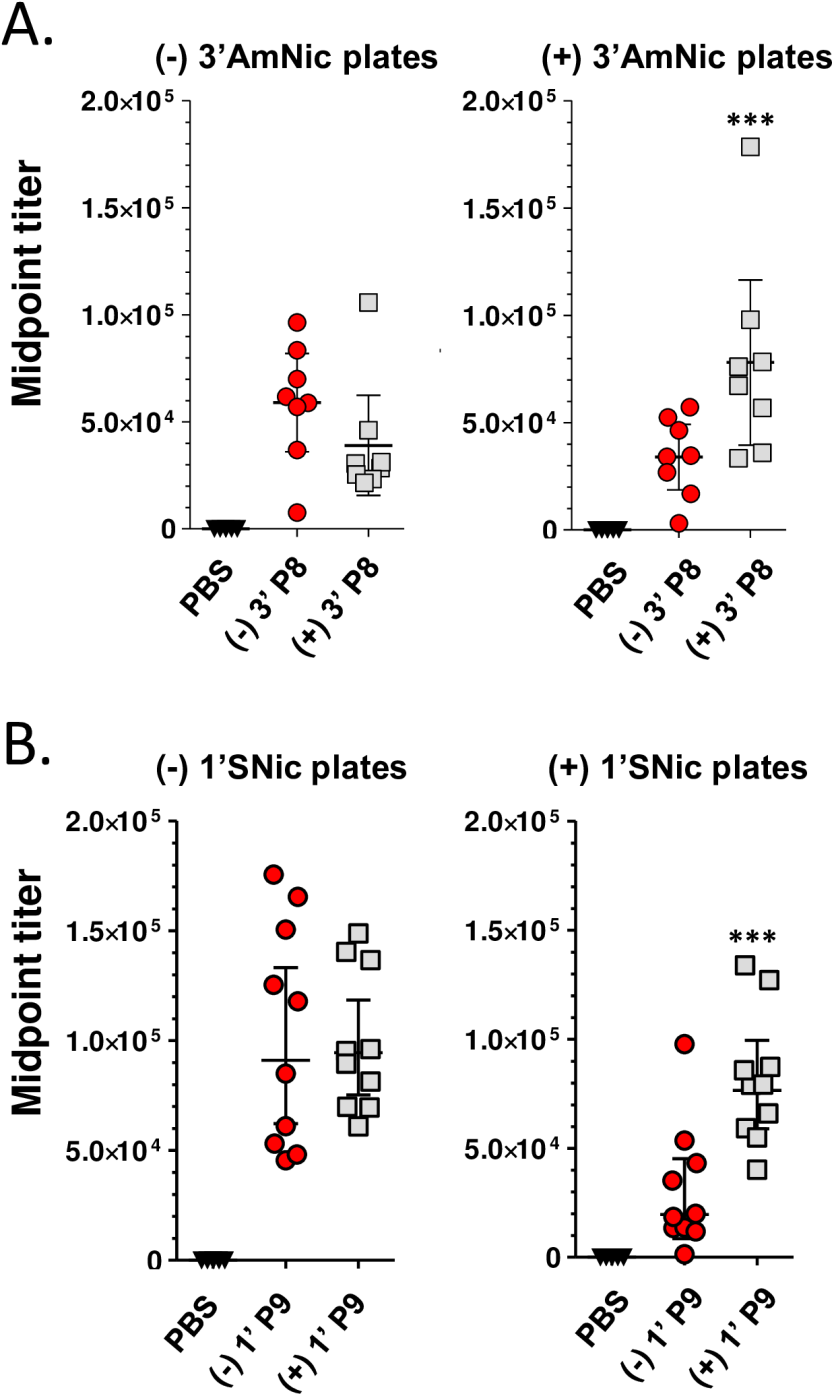
Antibody titers induced by enantiopure 3’-AmNic and 1’-SNic haptens. CD-1 female mice (n = 8) received a prime boost boost immunization with either P8 peptide (5 µg) conjugated to (-) 3’ or (+) 3’AmNic haptens (A); or with P9 peptide (5 µg) conjugated to (-) 1’ or (+) 1’SNic haptens (B). Serum was collected 56 days later and assayed by ELISA using plates coated with the reciprocal enantiopure haptens conjugated to BSA. Comparisons between groups were conducted by unpaired t-test. ***p<0.001.

**Fig 3:**
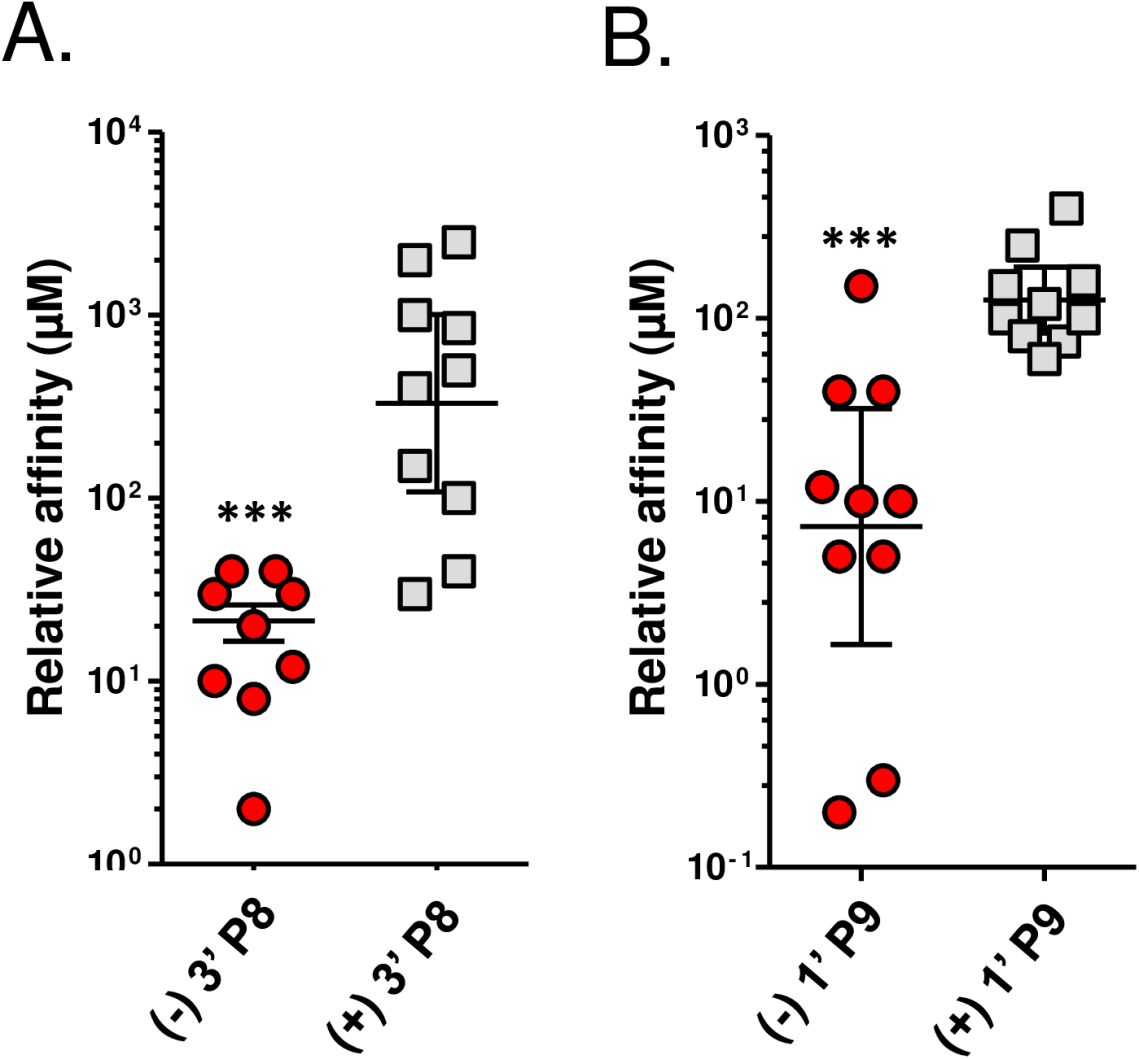
Relative nicotine binding affinities improve when using natural (-) enantiomers of two nicotine haptens. Day 56 serum was subjected to a competitive ELISA assay using serially diluted (-) nicotine as the competitor. Comparisons between groups were conducted by unpaired *t*-test. ***p<0.001.

**Fig 4:**
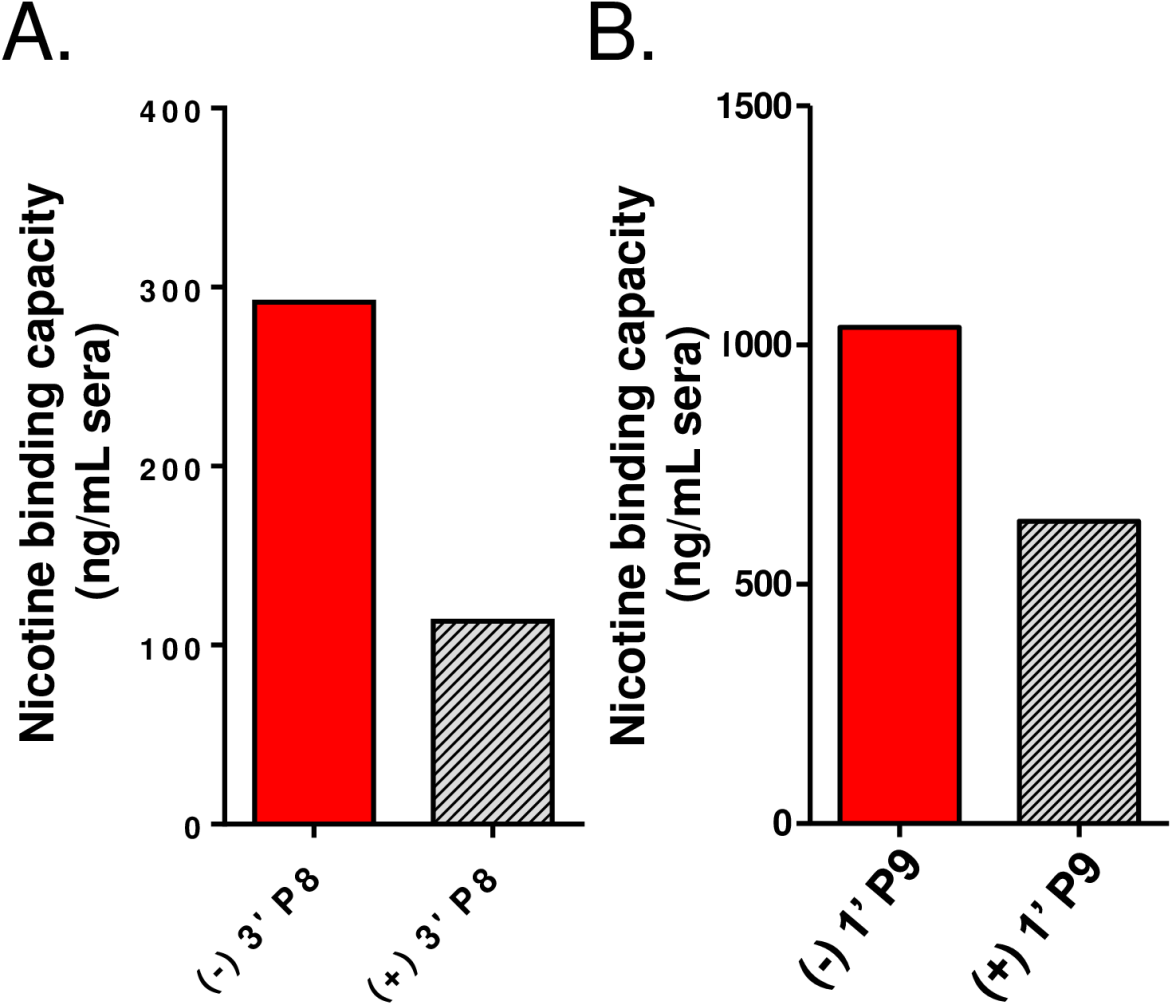
Serum nicotine binding capacities improve when using natural (-) enantiomers of two nicotine haptens. Day 56 serum was collected and pooled from mice immunized with 5 µg (-) 3’ P8 or (+) 3’ P8 **(A)** or 5 µg (-) 1’ P9 or (+) 1’ P9 **(B).** Nicotine binding capacities were determined by equilibrium dialysis.

It has been reported that Ab responses to natural (-) nicotine were substantially improved in rats following immunization with a tetanus toxoid carrier and an enantiopure (-) 3’AmNic hapten [23]. To test how hapten chirality might influence vaccine performance, racemic 3’AmNic and 1’SNic haptens were separated into (-) and (+) enantiomers and conjugated, respectively, to P8 and P9 peptides (see Methods). CD-1 mice received a prime-boost-boost immunization (5 ug antigen plus adjuvant) and day 56 sera were assayed for binding specificity to (-) and (+) haptens. As indicated in Fig 2A, the Abs induced with (-) 3’ P8 and (+) 3’ P8 recognized both chiral forms of 3’AmNic, although cross-enantiomer binding was lower for both immunogens. This difference was statistically different for (-) 3’ P8 serum on (+) 3’AmNic-coated plates, but only qualitatively different for (+) 3’ P8 serum on (-) 3’AmNic-coated plates. Abs induced by (-) 1’ P9 and (+) 1’ P9 peptides also recognized both enantiomers; binding of antisera from (-) 1’ P9 immunized mice to (+) 1’SNic was diminished but those induced with (+) 1’ P9 vaccine bound both (-) and (+) 1’SNic equally well (Fig 2B). In addition to specificity of enantiomer binding, the relative binding affinity of these antisera for native (-) nicotine was measured by competitive ELISA (Fig 3), and the overall serum binding capacity for (-) nicotine was determined by equilibrium dialysis (Fig 4). As indicated, the relative binding affinity and nicotine binding capacity was markedly better for the Abs induced by the (-) 3’AmNic and (-) 1’SNic immunogens, relative to the (+) enantiomers.

**Fig 5:**
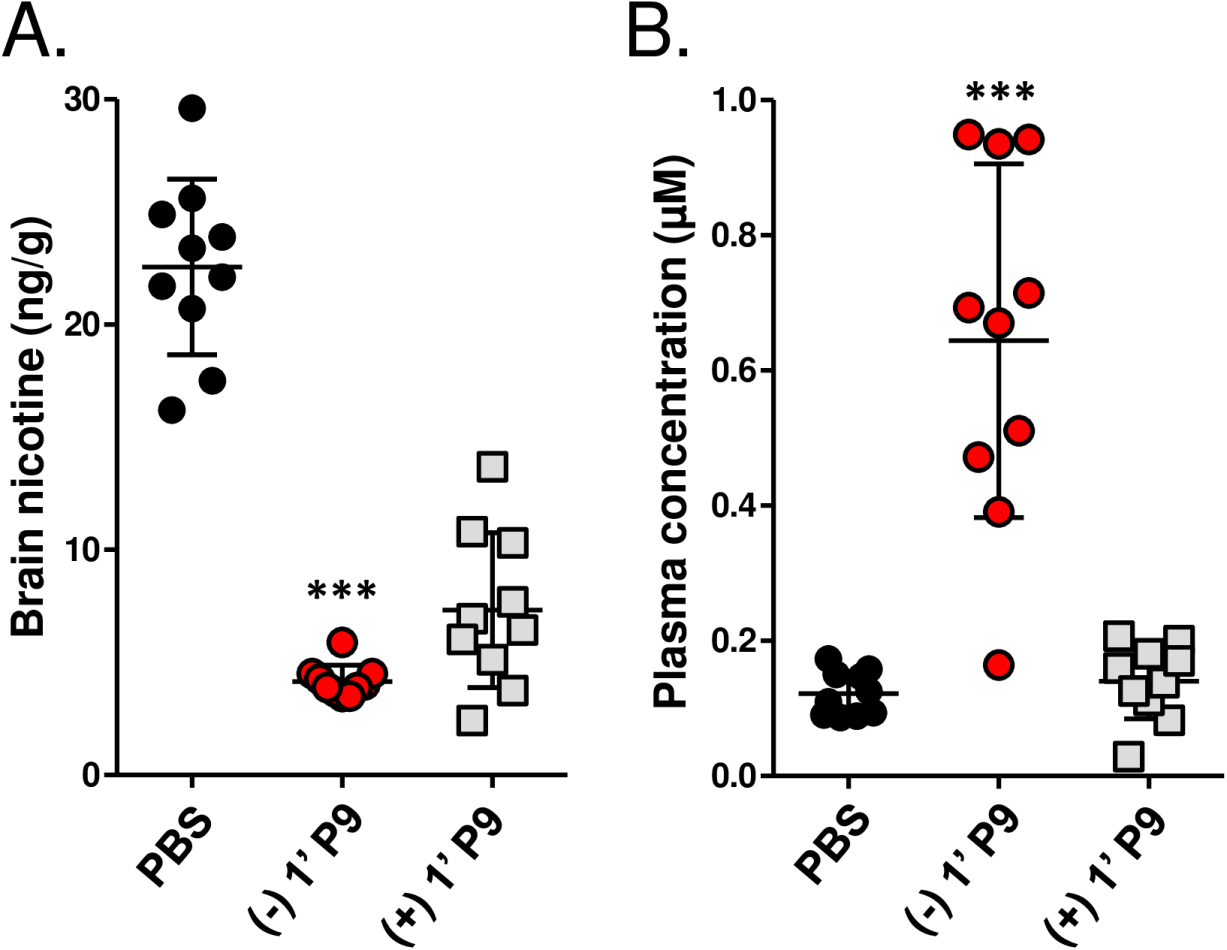
Vaccine efficacy is improved by using the natural (-) enantiomer of hapten 1’-SNic. Mice were immunized with 5 µg (-) 1’ P9 or (+) 1’ P9 on days 0, 21 and 42. On day 70, mice were injected with an amount of nicotine tartrate equivalent to three cigarettes (0.05 mg/kg). Mice were sacrificed after 5 minutes and nicotine levels were measured in brain tissue **(A)** and sera **(B)** by LC-MS/MS. Comparisons between groups were conducted one-way ANOVA. ***p<0.01 between vaccinated groups.

To test for a functional difference between hapten enantiomers, mice immunized with (-) 1’ P9 and (+) 1’ P9 were subjected to a nicotine brain/serum partitioning study on day 70. Nicotine (0.05 mg/kg, 3 cigarette equivalents) was injected intravenously; 5 minutes later, blood and brain samples were collected and nicotine content was quantified by LC-MS/MS. When compared to PBS immunized mice, <20% of the infused dose of nicotine was detected in the brains of (-) 1’SNic immunized mice (Fig 5A), with most the nicotine being retained in sera (Fig 5B). In contrast to this, brain nicotine levels were greater and more varied in (+) 1’SNic animals and their nicotine blood levels were indistinguishable from the control PBS group. Collectively, these data confirm that hapten chirality influences Ab specificity and that enantiopure vaccines should be used to maximize Ab responses to native (-) nicotine.

### Bivalent vaccines enhance functional Ab responses in an additive manner

**Fig 6:**
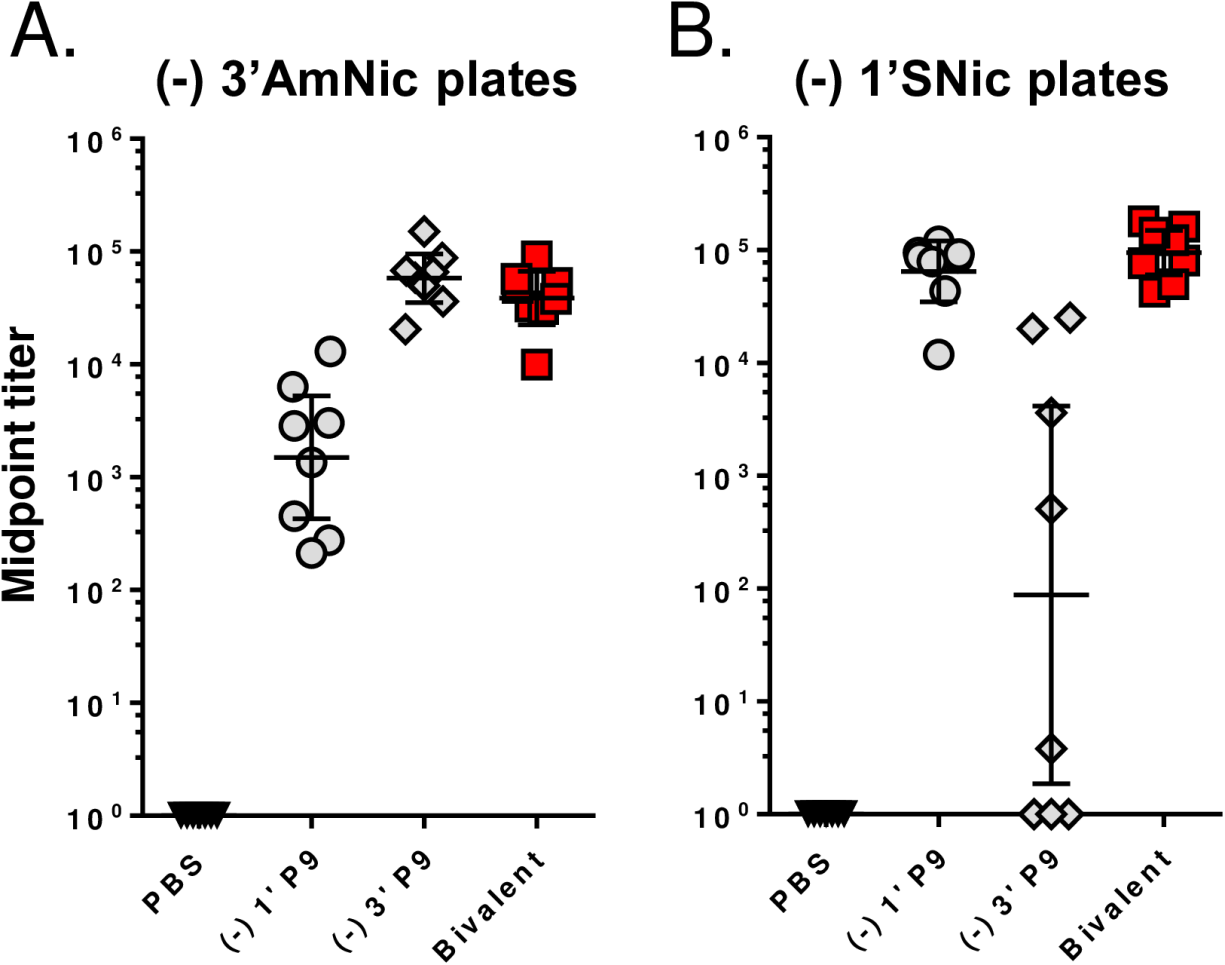
Antibody cross-reactivity between 1’-SNic and 3’-AmNic monovalent and bivalent vaccines. Serum was collected 56 days after priming from mice immunized with: (1) 10 µg (-) 1’ P9, (2) 10 µg (-) 3’ P9 or (3) 5 µg (-) 1’ P9 + 5 µg (-) 3’ P9. Sera titers were assayed by ELISA using plates coated with (-) 3’-BSA **(A**) or (-) 1’-BSA (**B)**.

**Fig 7:**
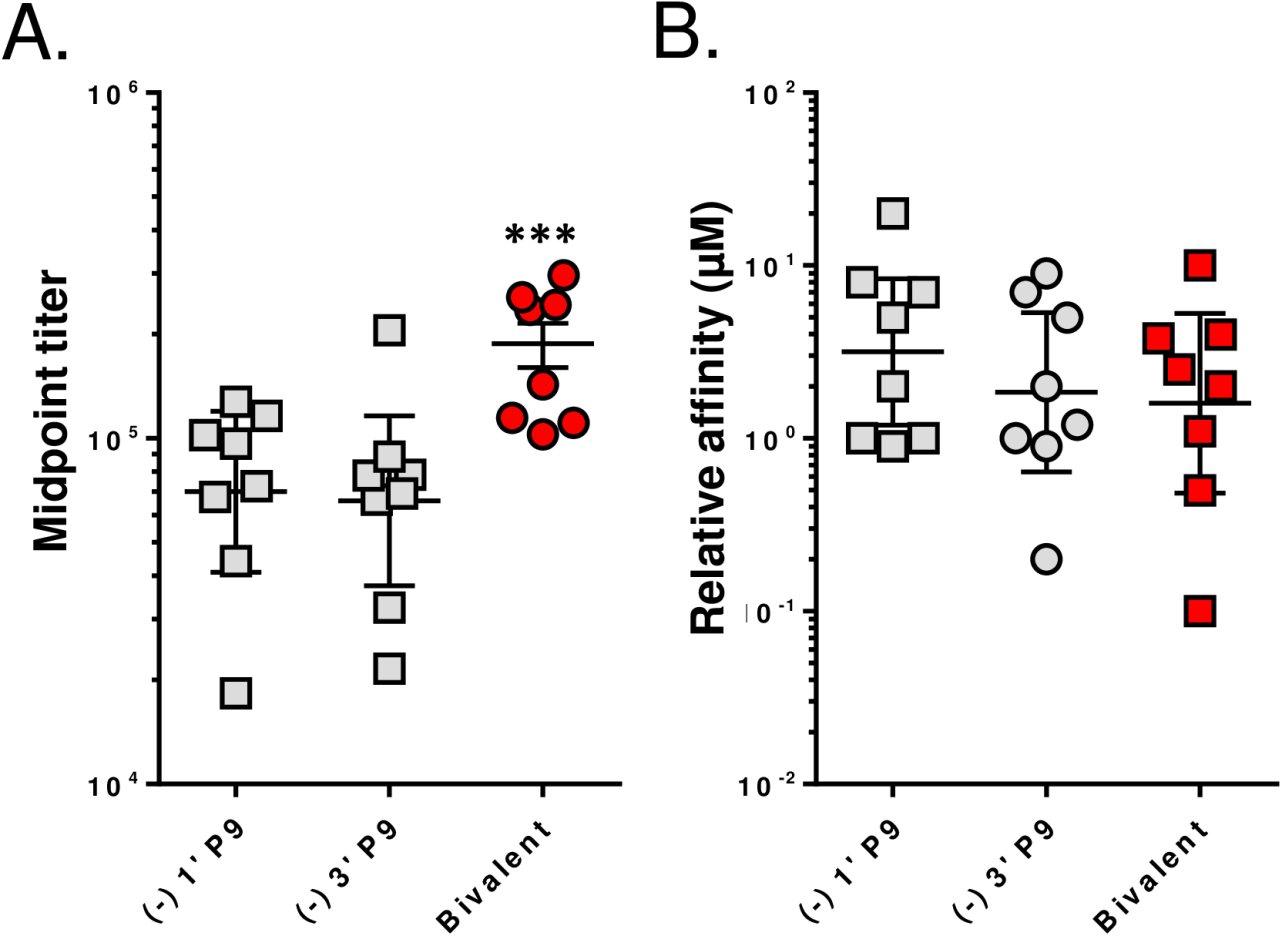
A bivalent nicotine vaccine stimulates an additive increase in antibody titers and equivalent avidities compared to dose-matched monovalent vaccines. Serum was collected 56 days after priming from mice immunized with: (1) 10 µg (-) 1’ P9, (2) 10 µg (-) 3’ P9 or (3) 5 µg (-) 1’ P9 + 5 µg (-) 3’ P9. Sera titers **(A)** and affinities **(B)** were assayed by ELISA using plates coated with a 50/50 mixture of (-) 1’ BSA and (-) 3’ BSA. Comparisons between groups were conducted by one-way ANOVA. ***p<0.01.

Previous work has shown that structurally-distinct haptens, which differ in linker design and attachment points to the nicotine molecule, can activate different B cell clones and that vaccines formulated with 2 or 3 haptens increased functional Ab concentrations relative to a monovalent vaccine [25–28]. To test this strategy, mice received a prime-boost-boost immunization with 10 µg of the enantiopure (-) 1’ P9 or (-) 3’ P9 conjugates, or 10 µg of a bivalent formulation containing 5 µg of (-) 1’ P9 and 5 µg of (-) 3’ P9. To show that these haptens stimulate different B cell clones, we measured the degree of hapten cross-reactivity on ELISA plates coated with (-) 3’AmNic-BSA (Fig 6A) or (-) 1’SNic-BSA (Fig 6B). The Abs induced by the monovalent vaccines showed <10% cross-reactivity and the bivalent formulation stimulated titers comparable to the respective monovalent groups. To test whether this leads to an additive increase in anti-nicotine Abs, we incubated sera with ELISA plates coated with a 50:50 mixture of (-) 1’SNic and (-) 3’AmNic and observed a roughly twofold increase in total Ab production relative to the monovalent groups (Fig 7A). No differences in relative Ab binding affinity were detected between the monovalent and bivalent immunization groups (Fig 7B).

**Fig 8:**
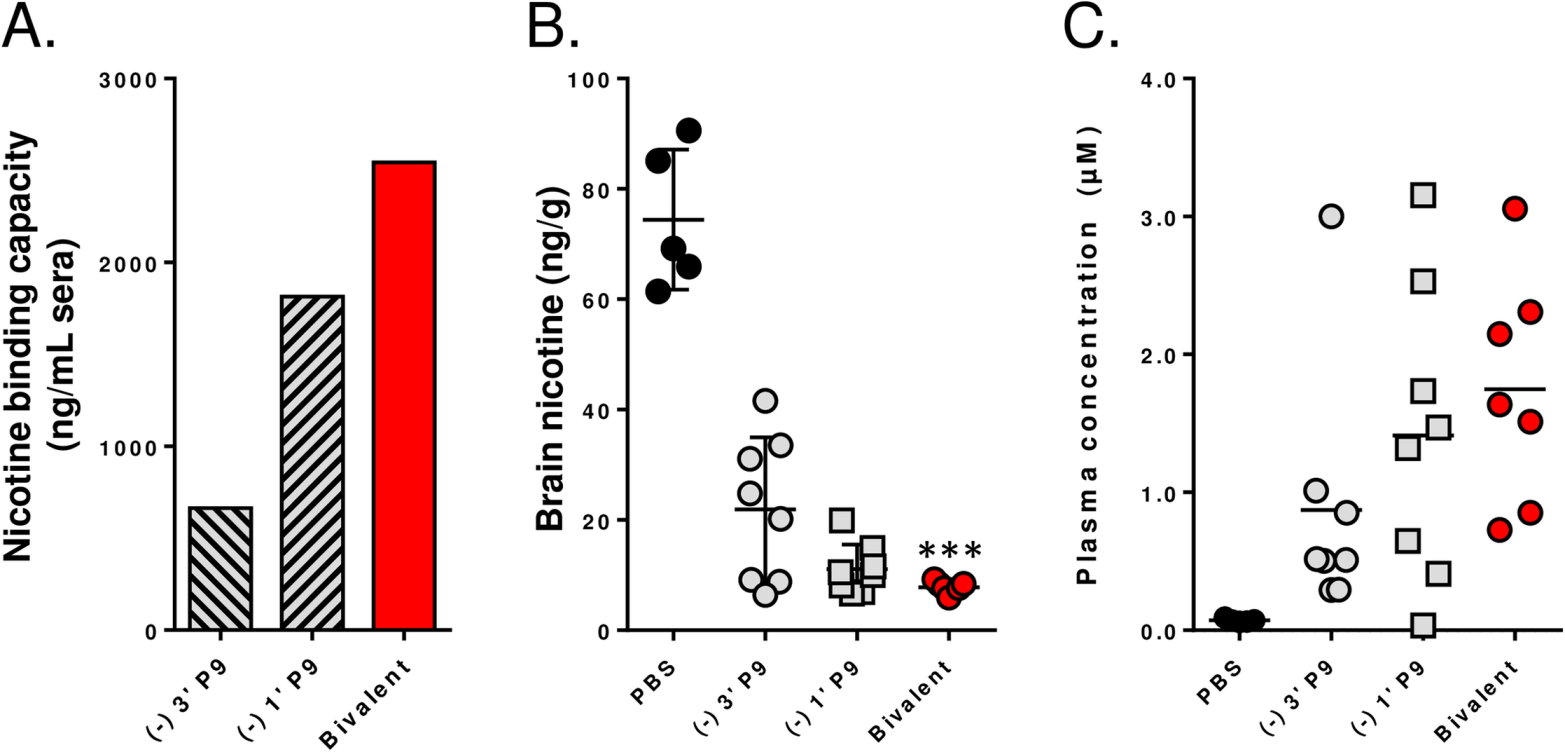
Functional antibody responses are improved with a bivalent vaccine. Plasma was collected on day 56 and pooled from mice immunized on days 0, 21 and 42 to determine nicotine binding capacity by equilibrium dialysis (A). Fourteen days later, mice were injected with an amount of nicotine tartrate equivalent to 12 cigarettes (0.2 mg/kg). Mice were sacrificed after 5 minutes and nicotine levels were measured on brain (B) and plasma (C) by LC-MS/MS.

To confirm that the bivalent formulation improves functional Ab concentrations, the serum nicotine binding capacity was measured (Fig 8A), and a second nicotine brain/blood partitioning experiment was performed, with a four-fold higher nicotine dose (0.2 mg/kg; 12 cigarette equivalents) than was used previously to better resolve functional differences between groups. The serum binding capacity for nicotine in the bivalent group was 2,560 ng/mL, which was equal to the sum of the binding capacities of the (-) 3’ P9 (665 ng/mL) and (-) 1’ P9 (1815 ng/mL) groups. It is worth noting that the binding capacity of the (-) 1’ P9 group in the bivalent study is roughly twofold higher than the (-) 1’ P9 group in the enantiomer experiment (ca. 1000 ng/mL). In-house dose-response studies showed that increasing the (-) 1’ P9 dose from 5 to 10 µg led to a doubling in Ab titers (data not shown); this increase in Ab titer is congruent with the increased binding capacity across studies. Correspondingly less nicotine accumulated in brains of mice immunized with the bivalent vaccine (7 ng/g) relative to the 3’-P9 (22 ng/g) and 1’-P9 (11 ng/g) monovalent vaccines (Fig 8B), and proportionally more nicotine was present in circulating sera (Fig 8C). Collectively, these results argue that a vaccine using two different nicotine haptens will independently activate populations of B cells resulting in an additive increase in functional Ab concentrations.

## Discussion

Nicotine vaccines tested in humans have failed to generate Ab responses capable of preventing nicotine from reaching the brain in a meaningful way [8,19], and a strong effort has been made to modernize conjugate vaccine technology. The hapten used in Phase III studies, 3’ AmNic, was a racemic mixture of (-) and (+) enantiomers, and recent studies report that nicotine binding capacities of immunized rat sera can be improved using enantiopure (-) 3’AmNic [23]. We tested this concept using enantiomer pairs of both 3’-AmNic and 1’-SNic haptens, and like the previous report, there was a marked increase in functional activity when the hapten shared the same chirality as naturally-occurring nicotine. In our study, only minor differences in binding specificity were detected between antisera induced with (-) and (+) haptens and (-) and (+) coated antigens, and cross-reactivity between (-) and (+) reactants was more apparent than measured by Lockner et al [23]. However, a significant difference in Ab binding affinity was observed in our experiments; antisera from (-) 1’SNic and (-) 3’AmNic groups had 17- and 38-fold stronger affinities than those from (+) 1’SNic and (+) 3’AmNic groups, respectively. Correspondingly, both (-) enantiomers induced nicotine binding capacities that were roughly twofold greater than their (+) counterparts. Furthermore, following an intravenous injection of nicotine, mice immunized with (-) 1’SNic retained significantly more nicotine in serum and nicotine accumulation in brain was decreased >80% relative to the PBS control. Conversely, sera nicotine levels of mice immunized with (+) 1’SNic were indistinguishable from the PBS control group and nicotine accumulation in brain was decreased by 65%. These data confirm that hapten chirality influences Ab recognition and argue that future clinical candidate vaccines should only use enantiopure haptens.

A second method for improving vaccine efficacy involves multivalent hapten formulations with different nicotine linkers and attachment sites that increase the breadth of antigen presentation and B cell activation. The linkers in 3'AmNic and 1'SNic differ in length, chemical make-up and attachment points on opposite sides of the nicotine pyrrolidine ring. Evidence that they engage different populations of Ig receptors was based on ELISA where cross-reactivity between antisera and the opposing hapten-coated plates was less than 10%. The notion that bivalency stimulates a complimentary increase in Ab production was reflected by the ~two-fold higher Ab titer in the bivalent group vs the monovalent groups and a concomitant additive increase in nicotine binding capacity. Importantly, the broadened Ab response in the bivalent group resulted in a statistically superior inhibition of nicotine accumulation in brain. As first reported by the Pentel laboratory [27], a vaccine containing 1'SNic, 3'AmNic and a third hapten with a linker attachment site at the 6 position of the pyridine ring (6-CMU-Nic), induced a functional Ab response that was greater than monovalent vaccines. Thus, it may be possible to formulate a multivalent vaccine containing a fourth or fifth hapten that presents the nicotine moiety to antigen receptors in a structurally distinct way that minimizes inter-hapten cross-reactivity. The use of coiled-coil peptide carriers seems particularly well suited for this approach because of their ease of manufacturing and downstream processing resulting in a reduced cost of goods. In conclusion, formulation of an enantiopure bivalent vaccine substantially increased Ab affinity and concentrations to levels that inhibited a nicotine dose equivalent to 12 cigarettes from reaching the brain. Given these promising results, it will be important to see how this technology translates from mice to larger species.
